# CUSTOMIZED GUIDE FOR FEMORAL COMPONENT POSITIONING IN HIP RESURFACING ARTHROPLASTY

**DOI:** 10.1590/1413-785220172502167422

**Published:** 2017

**Authors:** Chaturong Pornrattanamaneewong, Rapeepat Narkbunnam, Keerati Chareancholvanich

**Affiliations:** 1 Mahidol University, Faculty of Medicine, Siriraj Hospital, Department of Orthopedic Surgery, Bangkok, Thailand.

**Keywords:** Arthroplasty, replacement, hip/instrumentation, Femur neck, Imaging, three-dimensional, Surgery, computer-assisted, Prosthesis design

## Abstract

**Objective:**

To prove the accuracy of a customized guide developed according to our method.

**Methods:**

This customized guide was developed from a three-dimensional model of proximal femur reconstructed using computed tomography data. Based on the new technique, the position of the guide pin insertion was selected and adjusted using the reference of the anatomical femoral neck axis. The customized guide consists of a hemispheric covering designed to fit the posterior part of the femoral neck. The performance of the customized guide was tested in eight patients scheduled for total hip arthroplasty. The stability of the customized guide was assessed by orthopedic surgeons. An intraoperative image intensifier was used to assess the accuracy.

**Results:**

The customized guide was stabilized with full contact and was fixed in place in all patients. The mean angular deviations in relation to the what was planned in anteroposterior and lateral hip radiographs were 0.5º ± 1.8º in valgus and 1.0º ± 2.4º in retroversion, respectively.

**Conclusion:**

From this pilot test, the authors suggest that the proposed technique could be applied as a customized guide to the positioning device for hip resurfacing arthroplasty with acceptable accuracy and user-friendly interface. ***Level of Evidence IV, Cases Series.***

## RESUMO

**Objetivo:**

Comprovar a precisão de uma guia personalizada desenvolvida a partir de nosso método.

**Métodos:**

Esta guia personalizada foi desenvolvida a partir do modelo tridimensional da parte proximal do fêmur reconstruída usando dados de tomografia computadorizada. Com base na nova técnica, a posição de inserção do pino da guia foi selecionada e ajustada usando a referência do eixo anatômico do colo do fêmur. A guia personalizada consiste em um revestimento hemisférico projetado para encaixar na parte posterior do colo do fêmur. O desempenho da guia personalizada foi testado em oito pacientes que seriam submetidos à artroplastia total do quadril. A estabilidade da guia personalizada foi avaliada por cirurgiões ortopedistas. Para avaliar a precisão, usou-se um intensificador de imagem intraoperatório.

**Resultados:**

A guia personalizada foi estabilizada com contato total e foi fixada em todos os pacientes. Os desvios angulares médios com relação ao planejado nas radiografias anteroposteriores e laterais do quadril foram de 0,5º ± 1,8º em valgo e 1,0º ± 2,4º em retroversão, respectivamente.

**Conclusão:**

A partir deste teste piloto, os autores sugerem que a técnica proposta poderia ser aplicada como guia personalizada para o dispositivo de posicionamento para resurfacing em artroplastia de quadril com aceitável precisão e interface amigável. ***Nível de Evidência IV, Série de Casos*** .

## INTRODUCTION

Hip resurfacing arthroplasty (HRA) is an alternative to total hip arthroplasty (THA). The advantages of this procedure include preservation of the femoral bone stock,^1^ minimized dislocation rate,^2^ and improved range of motion.^3^ However, HRA is a technically demanding procedure and femoral neck fracture has been documented as the most common cause of early failure.^4^ This complication is related to varus malposition of the femoral component and superior notching of femoral neck.^5^ ^,^ ^6^

Accurate positioning of the femoral component has been reported in association with successful long-term outcomes.^7^ Optimal alignment traditionally is achieved using manual devices, and accuracy relies largely on visual inspection and the surgeon's experience. Computer assisted navigation can increase the accuracy of femoral guide pin insertion compared to conventional instrumentation.^8^ ^,^ ^9^ Nevertheless, it has distinctive disadvantages, including increased surgical time and cost.^8^

Patient-specific instrumentation for HRA is a novel device fabricated using rapid prototyping technology (RP). Computed tomography (CT) scanning provides individual 3D geometric anatomy data to construct a patient-specific instrument. The instrument is used to guide the position of pin insertion to avoid malpositioning of the femoral component. The most important reference axis for determining guide pin direction is the femoral neck axis (FNA). In clinical practice, determining the true FNA can still be problematic. 

Although several patient-specific guides (PSG) have been proposed in the literature and demonstrated utility with good accuracy,^10^ ^-^ ^14^ few studies state the method for defining true FNA.^11^ ^,^ ^14^ To our knowledge, the best-known technique was developed by Mahaisavariya et al.,^15^ who established a method for geometrical assessment of the proximal femur in three dimensions. This method uses CT images combined with reverse engineering to obtain the 3D geometry of the proximal femur. This technique can be applied to HRA in order to identify the true FNA. Consequently, the objectives of this study were to use this technique to design a PSG to assist in femoral component positioning in HRA, as well as assess the accuracy of this device.

## PATIENTS AND METHODS

**Figure 1:**
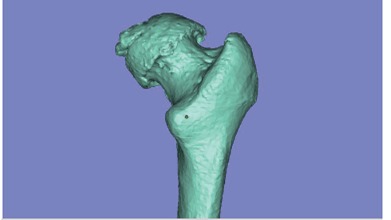
Three-dimensional graphic model of the proximal femur.

Between May and August 2011 we recruited eight patients scheduled for unilateral primary THA to participate in the study. Preoperative CT scans of the hip were performed with a 1-mm slice thickness. The axial cross-sectional images of the body were formatted into Digital Imaging and Communications in Medicine (DICOM) files and transferred to National Metal and Materials Technology Center (MTEC, National Science and Technology Development Agency, Pathumthani, Thailand). Medical imaging processing software (Mimics, Materialise N.V., Belgium) was used to convert the set of DICOM files to a three-dimensional image of hip. In the reconstruction process, the stack of DICOM files was sequenced in such the way that the relative proximal cross-section images were above the distal cross-sectional images. This allowed the femoral head to be oriented proximally to the femoral shaft. Each image was within the threshold range for Hounsfield unit (HU) values to capture bone density. Images of the proximal femoral region were separated from other bones using a region-by-region growing algorithm.^15^ The captured boundaries of the proximal femoral region were interpolated in a 3D computer aided design (CAD) model of the proximal femur, as illustrated in Figure 1.

PSG design can achieve success in HRA because it can precisely determine the true FNA. Nevertheless, if the femoral head was severely deformed, the mirror image technique from the contralateral side was used to calculate this axis. The engineers at MTEC developed a technique to derive true FNA as follows: the least square regression of ellipse and sphere was performed at the femoral neck region and femoral head, respectively. The centers of the ellipse and sphere together derived the line using the linear regression technique; this derived line was the true FNA. However, since is difficult to determine the correct cross-section plane at the femoral neck used for least square regression of the ellipse, the iteration of the aforementioned least square regression technique was performed until the FNA resulting from the current iteration was no more than ± 0.5º different from the FNA resulting from the previous iteration.^15^

**Figure 2:**
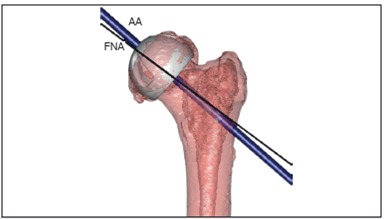
Adjusted axis (AA) to the direction of 5º valgus from the true femoral neck axis (FNA).

**Figure 3:**
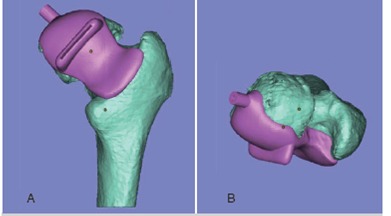
Design of patient-specific guide, (a) posterior and (b) superior view of proximal femur.

The guide pin was planned following the true FNA for 5 cases and plus additional overcorrection of 5 degrees valgus for 3 cases.

(Figure 2) The PSG was hemispherical to cover the femoral head, and was designed to be placed on the posterior part of the femoral head and neck. (Figure 3) In addition, the PSG contained the sleeve to control drilling direction, allowing a 3.2-mm diameter guide pin to be inserted through it. The primary goal of these devices was to securely fit the femoral neck, because of concerns related to the indistinct contours of articular cartilage in the CT images. The PSG was fabricated using a RP machine developed in our facility using an acrylate resin biocompatible for bone contact. The PSG was then polished and cleaned to remove residual particles, and sterilized using gamma radiation.

Before PSG was used in surgery, trials were performed in all cases. The PSG were tested to assess contact with the physical model of the proximal femur, stability of fixture, and contact clearance, as well as evaluate the direction of the guide after drilling. These assessments were made by the orthopedic surgeons as well as the engineers.

**Figure 4:**
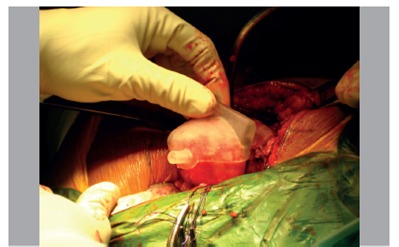
Intraoperative application of the patient-specific guide.

This study was approved by the institutional review board of Siriraj Hospital. All preoperative CT scans were done within 4 weeks prior to surgery. All surgical procedures were performed by the senior author (CK). The patient was placed in a lateral decubitus position, and a posterolateral approach of the hip was performed. After dislocation of the femoral head, the PSG was wrapped around the posterior part of femoral head and neck and locked in a stable snap-fit position. (Figure 4) The contact obtained at the neck portion and the stability of fixture was graded by the surgeons (full contact/unmovable, partial contact/unmovable, and partial contact/movable). A 3.2-mm-diameter guide pin was inserted via the pinhole and passed through the femoral neck. After removing the PSG, another end of the pin was cut at the level of 2 mm above the femoral head. The femoral head containing the pin was relocated. Pin alignment was assessed using the intraoperative image intensifier. For the anteroposterior (AP) view, the image intensifier was positioned perpendicular to the hip with femoral internal rotation of 15º. Without moving the image intensifier, the lateral view was obtained with a hip position of 45º flexion, 45º abduction, and 30º external rotation. After radiologic examination, the femoral head was dislocated again and the guide pin was removed. The remainder of the THA procedure was carried out as usual. No intra- or postoperative complications occurred in this series.

### Radiographic evaluation

**Figure 5:**
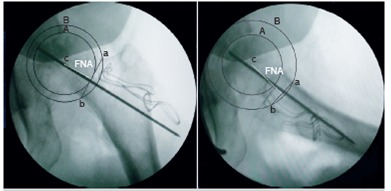
Radiographic assessment of guide pin position in anteroposterior (left side) and lateral (right side) radiographs of the hip; the center of the femoral head (C) is located within circle A. Another circle arc (B) was drawn using the center of the femoral head (C) as the center. Points a and b, where circle B intersects the deepest concave point of the femoral neck, are connected and defined as the transcervical line (ab line). The line drawn perpendicular to the transcervical line through the center of the femoral head (C) is represented as the femoral neck axis (FNA).

Two blinded assessors were assigned to evaluate the radiographs. From the AP view, we modified Muller's method^16^ to determine FNA as follows: the center of the femoral head was located with a circle. Reference points for the circle arc were the inferomedial and inferolateral border. The point of deepest concavity on the lateral border of femoral neck was marked. Another circle arc using the center of femoral head as the center was drawn. The points where the circle intersected the femoral neck were connected and defined as the transcervical line. Another line drawn perpendicular to the transcervical line through the center of the femoral head represented the FNA. This method was also applied in the lateral view, but used the anteroinferior and posteroinferior border to define the femoral head. (Figure 5)

The direction of the guide pin was compared to the FNA in either the AP or lateral radiographs. Deviations between these two lines were defined as angular deviations; varus, neutral or valgus angulation in the AP view and anteversion, neutral or retroversion in the lateral view. 

### Statistical analysis

Statistical analysis in this study was performed using SPSS version 18.0 software (SPSS Inc., Chicago, IL). Mean ± standard deviation (SD) was used to explain the descriptive statistics. Intraclass correlation coefficients (ICC) were used to assess the intra- and inter-rater reliability of radiographic measurements. 

## RESULTS

The characteristics of the patients and details of the assessed outcome are presented in Table 1. Mean patient age was 47.0 ± 12.1 years. The majority of the patients were diagnosed with osteonecrosis. The PSG was stabilized with full contact and was unmovable in all patients. The mean angular deviations from planning in the AP and lateral radiographs were 0.5º valgus ± 1.8º and 1.0º retroversion ± 2.4º directions, respectively. The ICC for inter-rater reliability was 0.83 and 0.91, while ICC for intra-rater reliability was 0.91 and 0.96 for angular deviation assessment in AP and lateral radiographs, respectively. 

**Table 1:** Patient characteristics and radiographic outcomes.

Number	Sex	Age (yrs)	Side	Diagnosis	Anteroposterior radiograph		Lateral radiograph	
					Aim	Angular deviation	Aim	Angular deviation
1	Female	26	Right	ON	0º	3º varus	0º	3º anteversion
2	Female	52	Left	ON	0º	1º valgus	0º	2º retroversion
3	Female	38	Right	PVNS	0º	neutral	0º	4º retroversion
4	Female	62	Right	OA	0º	neutral	0º	3º retroversion
5	Female	54	Left	OA	0º	3º valgus	0º	neutral
6	Female	58	Left	DDH	5º valgus	6º valgus	0º	3º retroversion
7	Female	48	Right	ON	5º valgus	7º valgus	0º	neutral
8	Male	38	Right	ON	5º valgus	5º valgus	0º	1º anteversion

ON = osteonecrosis, PVNS = pigmented villonodular synovitis. OA = osteoarthritis, DDH = developmental dysplasia of the hip.

## DISCUSSION

Various computer aided design and manufacturing technologies (CAD/CAM) were employed in this study to obtain the PSG used for HRA surgery. Proper selection of HU values in image processing along with reverse engineering technologies permit an accurate geometric model of the proximal femur to be constructed based on CT images. Determination of the true FNA based on the 3D proximal femur involves geometric approximation using various least square regressions, i.e. fit ellipse, fit sphere, and fit line. Until recently, the technologies described in this paper were not available for HRA, and FNA for most procedures was determined by the surgeon's skill, with varying results. These results may vary because the anthropometry of the Thai proximal femur, especially FNA, presents a wide range of values (110º-140º).^15^ ^)^ Therefore, the specific instrument for HRA presented in this study was reasonable. The PSG we developed is meant to facilitate the surgical procedure by precisely determining the true FNA and reducing trial and the use of radiography.

Unlike conventional radiographic imagery, three-dimensional CAD allows the true FNA to be determined. In order to demonstrate the accuracy of FNA determined through the PSG, we assessed the position during surgery and the angles measured in the AP and lateral views. The results show that the PSG has an acceptable coronal alignment accuracy of ±5º.^17^ In the sagittal alignment, although there was no evidence of acceptable alignment for anteversion, little angular deviation was shown from the study. 

True FNA is important for placing the femoral component in HRA. Most studies investigating PSG do not mention how to determine FNA;^10^ ^,^ ^12^ ^,^ ^13^ ^,^ ^17^ ^,^ ^18^ only one method, the so-called translucent cylinder method, appears in the previous literature. A translucent cylinder is created and superimposed on the 3D femoral model, and the position, orientation, and size of this cylinder are adjusted to identify FNA and neck diameter.^11^ ^,^ ^14^ Using the translucent cylinder method, Kunz et al.^11^ reported an angular deviation of 1.14º in coronal alignment and 4.49º in sagittal alignment. Du et al.^14^ assessed reports of angular deviation after using PSG with translucent cylinder method were displayed, and the results concentrated on the stem-shaft angle (SSA) difference between PSG and conventional instruments. 

Different PSG designs and surgical approaches have been proposed in the literature. (Table 2) Most authors used the PSG via the posterior approach to the hip. Acceptable coronal alignment was demonstrated in most studies,^10^ ^-^ ^14^ ^,^ ^18^ except for Olsen et al.,^17^ who reported an angular deviation of 6.4 ± 2.9º in the coronal plane. In the sagittal plane, a maximum angular deviation of 4.49º was reported using 3D CT navigation assessment.^11^

**Table 2:** Comparison with previous studies investing the accuracy of patient-specific guides.

Study	Published year	Subjects	Surgical approach of the hip	Outcome measurement	Angular deviation (mean ± SD)	
					Coronal plane	Sagittal plane
Kunz et al.^11^	2010	45 HRA	Anterolateral	CT navigation	1.14º	4.49º
Raaijmaakers et al.^12^	2010	5 THA	Anterolateral	Optical scan	Maximal 2.9º	
Zhang et al.^13^	2011	10 HRA	Posterior	Image intensifier	1.3 ± 1.0º	NA
Audenaert et al.^10^	2011	5 cadavers	Posterior	CT	4.05 ± 1.84º	
Du et al.^14^	2013	16 HRA	Posterior	Plain radiographs	NA	
Kitada et al.^18^	2013	12 synthetic femoral models	Posterior	CT	2.5 ± 2.4º	1.5 ± 2.3º
Olsen et al.^17^	2009	6 cadavers	Direct lateral Posterior	Plain radiographs	6.4 ± 2.9º	1.0 ± 0.4º
Current study		8 THA	Posterior	Image intensifier	0.5 ± 1.8º	1.0 ± 2.4º

SD = standard deviation, HRA = hip resurfacing arthroplasty, THA = total hip arthroplasty. CT = computed tomography, NA = not applicable.

In the current study, the authors developed the PSG according to the true FNA obtained from CAD in conjunction with various regression techniques. Compared to other studies, the PSG we developed can be applied with good stability and provides acceptable accuracy in guide pin placement for HRA. (Table 2) Nevertheless, there are several limitations in this study; first, this study is preliminary and was only conducted in a small group of THA patients. Future studies should investigate the use of this device in HRA in larger groups. Second, PSG was designed only for the posterior approach. During surgery, damage to the vessels in the posterior capsule of the hip may cause avascular necrosis of the femoral neck, resulting in femoral neck fracture.^19^ Finally, the authors assessed the alignment using an image intensifier. Although we try to control the position of hip and leg, some imaging error may occur. Intraoperative CT scanning is the best option in this situation, but is not available in our institute.

## CONCLUSION 

This study presents the use of CAD/CAM in conjunction with various least square regression techniques to determine true FNA and develop and fabricate a PSG for femoral component positioning in HRA. The initial results from eight patients using CT based PSG are encouraging. The shape of the PSG was applied to the femoral neck and a secure fit was obtained, and accurate guide pin insertion using this device was verified.
